# Core decompression combined with bone marrow mononuclear cells in the treatment of femoral head necrosis: a systematic review and meta-analysis

**DOI:** 10.1097/JS9.0000000000001625

**Published:** 2024-07-11

**Authors:** Ying Zhu, Peiyuan Tang, Hua Chai, Wenbo Ma, Yangbin Cao, Han Tan, Bin Lin, Wenfeng Xiao, Ting Wen, Yusheng Li

**Affiliations:** a Department of Orthopedics, Xiangya Hospital, Central South University, Hunan; b National Clinical Research Center for Geriatric Disorders, Xiangya Hospital, Central South University, Changsha; c Xiangya School of Medicine, Central South University, Changsha, China

**Keywords:** bone marrow mononuclear cells, core decompression, femoral head necrosis, meta-analysis

## Abstract

**Background:**

The effectiveness of bone marrow mononuclear cells (BMMCs) combined with core decompression (CD) in the treatment of femoral head necrosis is controversial. The purpose of this study was to conduct a meta-analysis and systematic review of the evaluation of BMMCss combined with CD in the treatment of femoral head necrosis and to compare the therapeutic effect of this method with that of CD alone so as to provide a basis for subsequent research and clinical treatment.

**Methods:**

We conducted detailed searches across four databases in Embase, PubMed, Web of Science, and the Cochrane Library (up to October 2023), including eight studies with a total of 370 participants and 491 hip cases. This meta-analysis followed the Preferred Reporting Project (PRISMA) guidelines. Review Manager 5.4 was used to summarize and analyze the outcome indicators and the quality and reliability of the MAs were graded against a Measurement Tool to Assess Systematic Reviews 2 (AMSTAR 2).

**Results:**

Eight studies were included in the inclusion criteria. The results of meta-analysis showed that the therapeutic effect of CD combined with BMMC on visual analog scale was better than that of CD alone [mean difference (MD)=−5.32, 95% confidence interval (CI): −9.90, −0.74, *P*=0.02, *I* ^2^=98%], and there was no statistically significant difference between CD combined with BMMC and CD alone in the treatment of Harris hip score (MD=2.73, 95% CI: −2.63, 8.09, *P*=0.32, *I* ^2^=82%). We conducted a sensitivity analysis. The results showed that the CD joint BMMC treatment effect on the Harris hip score is superior to the single CD (MD=5.57, 95% CI: 1.94, 9.20, *P*=0.003, *I* ^2^=0%), both no significant differences in visual analog scale (MD=0.47, 95% CI: −1.74, 0.79, *P*=0.46, *I* ^2^=83%).

**Conclusion:**

In this study, we found that CD combined with bone marrow monocyte therapy improved femoral head necrosis better than CD alone.

## Introduction

Highlights

- Core decompression (CD) combined with bone marrow monocyte therapy can improve femoral head necrosis better than CD alone.
- Our study is the first meta-analysis so far to summarize the effectiveness of bone marrow mononuclear cells (BMMCs) combined with CD in the treatment of femoral head necrosis and compare it with CD alone.

Osteonecrosis of the femoral head (ONFH) is a progressive pathological process of hip injury, the main cause of which is vascular necrosis due to disturbance of blood circulation, as well as alcoholism, heavy use of corticosteroids and steroids, disorders of lipid metabolism, certain inflammatory or autoimmune diseases, sickle cell anemia, organ transplantation, viral infection, etc. These factors may lead to the local destruction of the femoral head^1,2^. Interrupted or impaired blood supply can lead to cell death, bone marrow composition changes, and trabecular microfractures of the femoral head, leading to structural changes and dysfunction of the femoral head. ONFH mainly affects young people and has a relatively high incidence. The clinical manifestations of patients are hip pain and limited flexion and extension activities. When the disease progresses to the advanced stage, it will cause femoral head collapse, which has a high disability rate and is difficult to cure^3^. At present, there is also a lack of effective clinical treatment. The current method is to restore the joint function of patients through total hip replacement, but this technology has more complications, and its service life is limited, and the probability of needing repair is large, which will bring huge economic and psychological burdens to patients. Finding a safe and effective method to treat femoral head necrosis and prevent its further development has been the focus of research^4^.

CD is the most commonly used method to treat femoral head necrosis. It can not only reach the necrotic bone lesion through drilling, release intramedullary pressure but also promote bone formation and blood supply reconstruction of necrotic femoral head and reshape bone structure. However, studies have also shown that 37% of patients treated with CD will develop femoral head collapse, which may be because the bone defect caused by CD will cause damage to the mechanical support structure of the femoral head, increasing the risk of late iatrogenic femoral head collapse. Bone transplantation is considered to be a method to make up for this defect of CD^5–8^. Currently, the mainstream bone transplantation includes autogenous bone transplantation (ABG), biomaterial bone transplantation (BBG), bone transplantation combined with bone marrow transplantation (BG+BM), and free vascular bone transplantation (FVBG)^4^. In bone marrow transplantation, BMMC are believed to induce angiogenesis and bone formation in ischemic tissue, and treatment of ONFH can reduce pain, improve hip function, and delay disease progression. Li *et al*.^9^ compared isolated monocytes with untreated bone marrow injection and found that hip function was significantly improved in both groups. In recent years, some studies have suggested that the combined treatment of BMMCs and CD may be more effective for ONFH than that of CD alone^10,11^. However, some studies have shown that monocyte combined with CD has no significant effect on ONFH, and its safety and effectiveness are still controversial. We hypothesize that bone marrow monocyte combined with CD can improve femoral head necrosis better than CD alone. Therefore, we conducted a meta-analysis of relevant literature, aiming to analyze whether the treatment effect of BMMCs combined with CD is better than that of CD alone in ONFH, so as to provide a basis for subsequent clinical research and selection of better treatment methods.

## Methods

This systematic review and meta-analysis was conducted in accordance with the methodological guidelines of the Cochrane Handbook of Systematic Review and PRISMA^12,13^ (Preferred Reporting Procedure for Systematic Review and Meta-Analysis Guidelines) (Supplementary Material S1, Supplemental Digital Content 1, http://links.lww.com/JS9/D505) and has been registered on the Prospero website.

### Search strategy

Searches were conducted in four databases, PubMed, Embase, Web of Science, and Cochrane Library, up to October 2023, including relevant randomized controlled trials and nonrandomized controlled trials, and there were no regional or publication date restrictions on the literature searched. The specific retrieval process and details can be referred to Supplementary Material S2, Supplemental Digital Content 2, http://links.lww.com/JS9/D506.

### Eligibility criteria

The PICOs question served as the basis for the eligibility criteria for the systematic review and meta-analysis. P: patients with ONFH. I: CD combined with BMMCs was used to treat femoral head necrosis. C: CD was used to treat femoral head necrosis. O: Harris hip score (HHS) and visual analog scale (VAS). S: randomized controlled trials, cohort studies, and case series studies.

Inclusion criteria: Patients with osteonecrosis of the femoral head. The degree of femoral head necrosis was consistent with the Association Study circulating Bone tissue (ARCO) stage. No restrictions were made on the ethnicity, sex, or nationality of patients. Contact follow-up is available to confirm any complications and patient-reported outcomes.

Exclusion criteria: Necrosis of the femoral head due to active infection and coagulation dysfunction. Literature with no available data or duplicate data. Animal studies and other nonclinical trials. Review and conference papers. Non-English literature. The follow-up time did not reach 1 year.

### Data extraction and quality assessment

The two authors independently extract and sift the data and evaluate the quality of the article, and in case of disagreement, consensus is reached through joint discussion^14^. Information such as author, country, number of patients, number of hip joints, average age, sex ratio, and results were extracted from the eligible articles. The main outcome indicators extracted in this study included VAS and HHS. The quality of cohort and case studies was assessed using the Newcastle-Ottawa Scale, while random controlled trials were assessed using the Cochrane Collaborative Assessment Risk of Bias tool^12^. We also used Assess Systematic Reviews 2 (AMSTAR 2)^15^ to assess the quality and reliability of the MAs covered in this meta-analysis.

### Statistical analyses

Review Manager 5.4 was used to summarize and analyze the extracted data, and a *P* value less than 0.05 was considered statistically significant. For continuous variables, mean difference (MD) and 95% confidence interval (CI) were used as evaluation indexes, and *I* ^2^ statistics and q statistics were used to judge the degree of heterogeneity of the included studies. If *I* ^2^>50% is considered as high heterogeneity, a random effects model should be adopted. The *I* ^2^<50% indicates good homogeneity, and the fixed-effect model can be used^16^. If heterogeneity is too large, sensitivity analysis is used to analyze the source of heterogeneity.

## Results

### Search results

**Figure 1:**
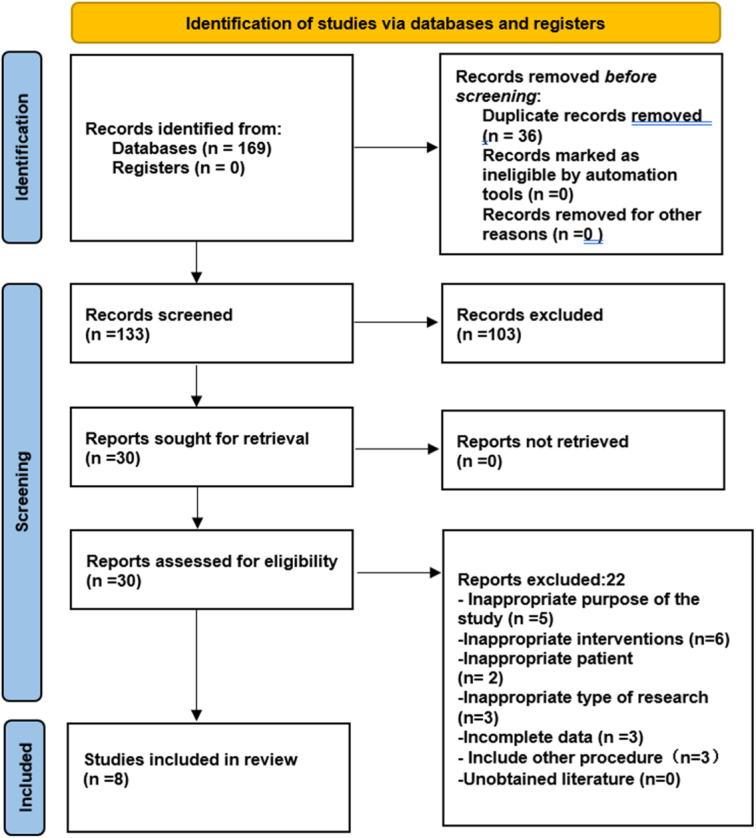
The Preferred Reporting Items for Systematic Reviews and Meta-analysis (PRISMA) flow diagram to show study selection.

A total of 169 literatures were obtained from the four databases at the beginning, and 36 duplicates were eliminated. After reading the title and abstract, the remaining 30 literatures were screened according to the established inclusion and exclusion criteria. Finally, by reading the full text, we excluded 22 studies that included inappropriate interventions, comparisons, etc. (Supplementary Material S3, Supplemental Digital Content 3, http://links.lww.com/JS9/D507), and included eight studies. Figure 1 depicts the literature screening process.

### Study characteristics

A total of eight articles were included in this meta-analysis, including three randomized controlled studies^1,17,18^, one cohort study^19^, and four case series studies^20–23^. Table 1 lists the basic information for all included studies. Of the 370 participants in all studies included, 277 were male and 90 were female in seven studies except for one study that did not have a male to female ratio^17^. All eight studies were followed up for at least 2 years. We also separately summarized the conclusions of the included studies. Gangji and Hauzeur^17^ found that CD and implantation of autologous BMMCs could significantly improve the Lequesne index, WOMAC index, and deterioration degree of the hip joint of patients, and implantation of BMMCs may be a safe and effective method for the treatment of early necrosis of the femoral head. Liang *et al*.^20^ believe that CD combined with autologous PRP and BMMC transplantation can effectively reduce the collapse rate of the femoral head in patients with ARCO Stage II and improve their hip joint function, which is A safe and effective method for treating nontraumatic ONFH in ARCO Stage II–IIIA. Liu *et al*.^21^ concluded that disease type is an important risk factor for BMMC transplantation combined with CD in the treatment of femoral head necrosis. In another study, Liu *et al*.^22^ found that CD combined with BMMC porous hydroxyapatite composite bone fillers can significantly improve hip function, reduce patient pain, and prevent femoral head collapse, which is of clinical significance for early femoral head necrosis. Sen *et al*.^18^ concluded that bone marrow monocyte infusion significantly improved clinical outcomes and hip survival and was more satisfactory for patients at higher risk for progressive lesions. Wang *et al*.^19^ suggested that concentrated autologous bone marrow transplantation containing mononuclear cells can significantly reduce hip pain, delay or avoid the need for hip replacement, and may be an option to prevent the progression of early femoral head necrosis, especially in the treatment of stage I–II nontraumatic femoral head necrosis. Yan *et al*.^23^ found that porous decompression combined with autologous BMMCs is a new method to treat avascular necrosis of the femoral head, and the earlier the necrosis time, the better the therapeutic effect may be. Tabatabaee *et al*.^1^ concluded that concentrated autologous bone marrow stem cells containing mononuclear cells combined with CD surgery was effective for early ONFH.

**Table 1:** Baseline characteristics of included literatures.

Authors	Year	Country	Age	Patients	Disease stage	Intervention	Hips	Sex (male/female)	Follow-up (months)	Outcome
Gangji and Hauzeur^17^	2005	Netherlands	NA	13	I/II	CD+BMMC	18	NA	24	WOMAC, Lequesne index
Liang *et al*.^20^	2023	China	36.4±5.3	44	II–IIIA	CD+BMMC+PRP	44	36/8	41.7±3.9	VAS, HHS
Liu *et al*.^21^	2018	China	NA	148	I–IIIA	CD+BMMC	192	117/31	36	HHS
Liu *et al*.^22^	2013	China	38.0±4.9	34	IIIB/IIC	CD+BMMC	55	27/7	26.7 ± 8.0	VAS, HHS
Sen *et al*.^18^	2012	India	NA	40	I/II	CD+BMMC	51	27/13	24	HHS, MRI, Kaplan–Meier hip survival analysis
Wang *et al*.^19^	2010	China	37.5	45	I–IIIA	CD+BMMC	59	36/9	27.6±6.12	HHS
Yan *et al*.^23^	2006	China	46	28	I–V	CD+BMMC	44	15/13	24	HHS
Tabatabaee *et al*.^1^	2015	American	31±11.4	18	I/II/III	CD+BMMC	28	19/9	24	WOMAC, VAS, MRI

BMMC, bone marrow mononuclear cells; CD, core decompression; HHS, Harris hip score; NA, not access; VAS, visual analog scale; WOMAC, Western Ontario and McMaster Universities Osteoarthritis.

All studies included in this meta-analysis had Newcastle-Ottawa Scale scores between 8 and 9. GRADE evaluation results showed that the overall quality of the study was very low (Supplementary Material S4, Supplemental Digital Content 4, http://links.lww.com/JS9/D508).

### The results of meta-analysis

#### Visual analog scale

**Figure 2:**
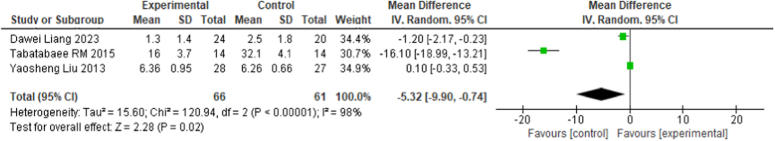
Forest plots of visual analog scale.

**Figure 3:**
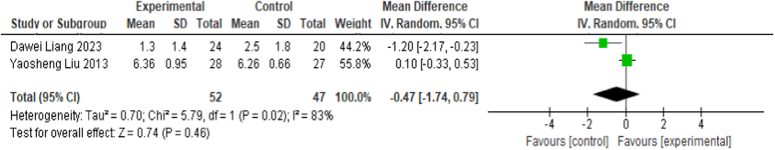
Forest plots of sensitivity analysis on the visual analog scale.

Three involving 96 participants, 127 cases of hip joint research report the CD BMMC treatment effect on the VAS^1,20,22^. Random effects model analysis showed that CD combined with BMMC was superior to CD alone in VAS (MD=−5.32, 95% CI: −9.90, −0.74, *P*=0.02, *I* ^2^=98%) (Fig. 2). Because the heterogeneity of the results was large, we performed a sensitivity analysis and found that heterogeneity was reduced only after the study of Tabatabaee *et al*.^1^ was removed (MD =−0.47, 95% CI: −1.74, 0.79, *P*=0.46, *I* ^2^=83%) (Fig. 3),and the results showed no significant difference in treatment effect between the two.

#### Harris hip score

**Figure 4:**
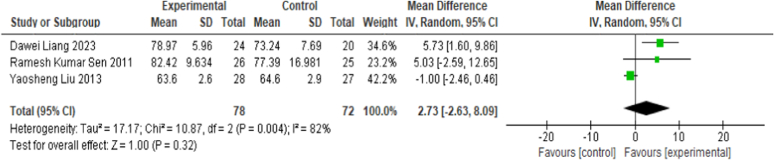
Forest plots of Harris hip score.

**Figure 5:**
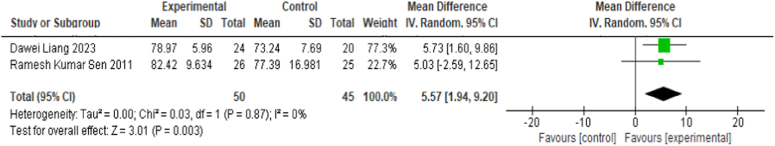
Forest plots of sensitivity analysis on Harris hip score.

Three involving 118 participants, 150 cases of hip joint research report CD combined with BMMC treatment on the effect of HHS^18,20,22^. The random effects model analysis used showed no statistically significant difference in HHS between CD alone and CD combined with BMMC (MD=2.73, 95% CI: −2.63, 8.09, *P*=0.32, *I* ^2^=82%) (Fig. 4). Since the high heterogeneity of meta-analysis results, we conducted a sensitivity analysis, and the results showed that after removing the study of Liu *et al*.^22^, the heterogeneity was significantly reduced, and the results indicated that CD combined with BMMC was superior to CD alone in HHS (MD=5.57, 95% CI: 1.94, 9.20, *P*=0.003, *I* ^2^=0%) (Fig. 5).

#### Pre–post on visual analog scale

**Figure 6:**
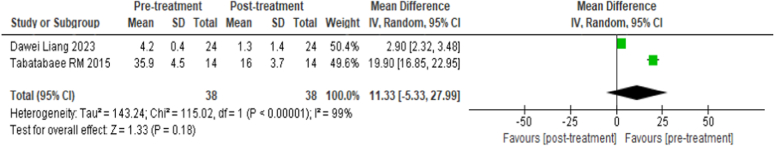
Forest plots of pre–post on the visual analog scale.

The pre-meta-analysis and post-meta-analysis included two studies involving 62 participants with 76 hip joints^1,20^. Random effects model was used to analyze the efficacy of CD combined with BMMC before and after treatment, and the results showed that there was no significant difference between CD combined with BMMC and VAS (MD=11.33, 95% CI: −5.3, 27.99, *P*=0.18, *I* ^2^=99%) (Fig. 6).

#### Pre–post on Harris hip score

**Figure 7:**
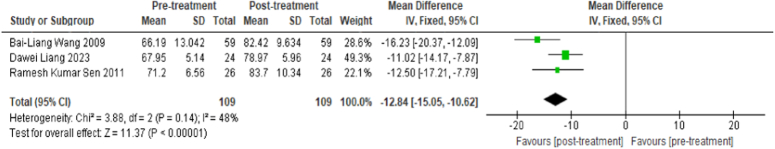
Forest plots of pre–post on Harris hip score.

The pre-meta-analysis and post-meta-analysis included three studies involving 129 participants, 218 hip joint^18–20^. A fixed-effect model was used to analyze the effects of CD combined with BMMC before and after treatment, and the results showed a significant association between CD combined with BMMC and improved HHS (MD=−12.84, 95% CI: −15.05, −10.62, *P*<0.00001, *I* ^2^=48%) (Fig. 7).

## Discussion

As a devastating disease, the prevalence of femoral head necrosis is increasing, and it usually affects younger patients^24^. A number of treatments have been developed to preserve the hip, with CD being the most common^25^. With the continuous development of research, it has been found that BMMC can promote angiogenesis and stimulate bone formation in the stage of bone healing^26^, and the method of BMMCs combined with traditional CD in the treatment of femoral head necrosis has also begun to attract the attention of scientists. However, due to the differences in the success rate and results reported by different reports, So the effectiveness of this treatment has always been controversial.

The meta-analysis of eight studies found that CD combined with BMMC was more effective than CD alone in terms of VAS score (MD=−5.32, 95% CI: −9.90, −0.74, *P*=0.02, *I* ^2^=98%). In terms of Harris score, there was no significant difference between CD combined with BMMC and CD alone (MD=2.73, 95% CI: −2.63, 8.09, *P*=0.32, *I* ^2^=82%). However, after sensitivity analysis, the results showed that after removing the study conducted by Liu *et al*.^22^, CD combined with BMMC was also superior to CD alone in terms of HHS (MD=5.57, 95% CI: 1.94, 9.20, *P*=0.003, *I* ^2^=0%). In addition, VAS score was not significantly improved (MD=11.33, 95% CI: −5.3, 27.99, *P*=0.18, *I* ^2^=99%), while Harris score was significantly improved (MD=−12.84, 95% CI: −15.05, −10.62, *P*<0.00001, *I* ^2^=48%).

In this study, there was considerable heterogeneity in the results of meta-analysis of VAS and HHS, for which we conducted a sensitivity analysis to find the cause of heterogeneity. We found that in the meta-analysis results of VAS score, the heterogeneity changed most significantly when Tabatabaee studies were removed. Therefore, Tabatabaee studies may be the main source of heterogeneity, which may be because the bone marrow stem cell transplantation containing BMMC was used in this study^1^. In the other two studies, isolated autologous BMMC was injected^20,22^, which means that different amounts of BMMC may have a certain impact on the therapeutic effect. However, in the results of the HHS meta-analysis, Liu’s study may be the main source of heterogeneity. After the removal of this study, not only was the heterogeneity significantly reduced, but also the HHS score of the CD-combined BMMC group was significantly improved, which may be due to the use of nano-hydroxyapatite/polyamide bone fillers in the BMMC in this study. Differences in implant materials may also affect the effectiveness of treatment. In addition, the age of the participants in the various studies was different, and age itself is a risk factor for femoral head necrosis, so age may also play a role in the source of heterogeneity^27^. Many studies have shown that BMMC is effective in the treatment of femoral head necrosis. Mao and colleagues found that the infusion of autologous bone marrow mesenchymal stem cells containing BMMC via the rotatory medial femoral artery could improve hip joint function, relieve patients’ symptoms, and delay the progression of ONFH, especially for the treatment of early ONFH^28^. Cai *et al*.^29^ concluded that combined transplantation of BMMC and umbilical cord mesenchymal stem cells was effective in treating ischemic femoral head necrosis (ANFH) without serious complications. Yamasaki *et al*.^30^ believed that BMMC transplantation may be a beneficial therapeutic method for bone repair in the case of femoral head necrosis. Kang *et al*.^31^ found that the combination of strontium-doped calcium polyphosphate and BMMC can enhance VEGF expression, which may promote the formation of blood vessels and trabecular bone and help prevent the progression of femoral head necrosis. Hernigou *et al*.^32^ concluded through a long-term follow-up study that injection of autologous concentrated bone marrow (including BMMC) combined with CD for treatment of femoral head necrosis was superior to CD alone in reducing the risk of femoral head collapse, delaying the need for hip replacement and improving the Harris score of ON patients. The results of our meta-analysis were consistent with the results of the above studies, which also proved the correctness of our hypothesis that bone marrow monocytes combined with CD may better improve femoral head necrosis compared with CD alone. We know that the pathogenesis of femoral head necrosis may be related to the following aspects: vascular occlusion or ischemia, changes in fat metabolism and fat embolism, intravascular coagulation^33^, so in order to effectively treat femoral head necrosis, it is very critical to restoring the blood supply to the lesion. Studies have shown that bone marrow monocytes can promote angiogenesis at osteonecrosis and improve blood circulation because they cannot only secrete growth factors, chemokines, and other influences on neighboring cells but also attract regenerative stem cells to the tissue repair site for the reconstruction of trabecular bone and microvascular of the femoral head^34,35^. Although the specific mechanism of BMMC promoting angiogenesis is still unclear, it is undeniable that it is beneficial to the recovery of femoral head necrosis. The combination of BMMC implantation and CD can better overcome the deficiency of clinical treatment of CD and reduce the bone defect caused by CD, which may be part of the reason why the treatment effect of BMMC combined with CD is better than that of CD alone. Overall, in the present study, CD combined with BMMC is effective in the treatment of femoral head necrosis. This method can reduce patients’ pain, delay hip collapse without serious complications, and is superior to CD therapy alone, which may be a safe and effective means for the subsequent treatment of femoral head necrosis, especially in the early stage.

This study also has the following limitations, which need to be further improved and perfected. The included studies had a small number of randomized controlled trials of CD combined with BMMC treatment, which may lead to publication bias. Some of the included studies had a small sample size, which may affect the effect of meta-analysis. There are certain differences in the follow-up time of the included studies, which may affect the scores of the detection indicators. For future research, we suggest the following aspects. First, researchers should conduct more randomized controlled trials. Second, in order to reduce bias, it is recommended that researchers increase the sample size of the study. Third, it is suggested that the clinical data of the participants should be fully improved during the design and implementation of the trial so as to compare the relevant characteristics of the experimental group and the control group. Fourth, for diseases with staging criteria, it is recommended that researchers try to compare subjects at the same stage together. Fifth, the duration of follow-up should be increased to reduce differences in the measurement outcomes when monitoring prognosis.

## Conclusion

In this study, we found that CD combined with bone marrow monocyte therapy improved femoral head necrosis better than CD alone.

## Ethical approval

Not applicable.

## Consent

Not applicable.

## Source of funding

This work was supported by National Key R&D Program of China (No. 2023YFC3603400), The Hunan Provincial Science Fund for Distinguished Young Scholars (No. 2024JJ2089), National Key R&D Program of China (2019YFA0111900), National Natural Science Foundation of China (No. 882072506,92268115, 82272611), National Clinical Research Center for Geriatric Disorders (Xiangya Hospital, Grant No. 2021KFJJ02 and 2021LNJJ05), National Clinical Research Center for Orthopedics, Sports Medicine and Rehabilitation (No. 2021-NCRCCXJJ-PY-40), Science and Technology Innovation Program of Hunan Province (No. 2021JJ31105), Hunan Provincial Innovation Foundation For Postgraduate (No. CX20230312 and No. CX20230308).

## Author contribution

Y.Z. and P.T.: conceived the study; Y.L. and T.W.: designed the study; Y.Z., T.W., and Y.L.: undertook the literature review and extracted the data; Y.Z. and T.W. coded the statistical analysis, figures, and appendix; and Y.Z.: interpreted the data and wrote the first draft of the manuscript. All authors read and approved the final manuscript.

## Conflicts of interest disclosure

The authors declare no conflicts of interest.

## Research registration unique identifying number (UIN)

This study has been registered at Prospero. Registration ID: CRD42023430975.

## Guarantor

Yusheng Li.

## Data availability statement

Data sharing is not applicable to this article.

## Provenance and peer review

Not commissioned, externally peer-reviewed.

## Supplementary Material

SUPPLEMENTARY MATERIAL
