# Supplementary material for: Core decompression combined with bone marrow mononuclear cells in the treatment of femoral head necrosis: a systematic review and meta-analysis
Source: Int J Surg. 2024 Jul 11;110(10):6763–70. doi: 10.1097/JS9.0000000000001625 (PMC11487039; doi:10.1097/JS9.0000000000001625)
Supplement: SUPPLEMENTARY MATERIAL [file js9-110-6763-s002.docx]

**1. Pubmed**

**(((((((((((((Femur Head Necrosis[Title/Abstract]) OR (Femur Head Necroses[Title/Abstract])) OR (Head Necrosis, Femur[Title/Abstract])) OR (Necrosis, Femur Head[Title/Abstract])) OR (Aseptic Necrosis of Femur Head[Title/Abstract])) OR (Necrosis, Aseptic, of Femur Head[Title/Abstract])) OR (Necrosis, Avascular, of Femur Head[Title/Abstract])) OR (Ischemic Necrosis Of Femoral Head[Title/Abstract])) OR (Femoral Head, Avascular Necrosis Of[Title/Abstract])) OR(Avascular Necrosis Of Femoral Head, Primary[Title/Abstract])) OR (Avascular Necrosis of Femur Head[Title/Abstract])) OR (Femur Head Necrosis[MeSH Terms])) AND ((Monocytes[MeSH Terms]) OR (((((((Monocytes[title/abstract])) OR (mononuclear cells[title/abstract])) OR (Mononuclear Leukocytes[title/abstract])) OR (Mononuclear Leukocyte[title/abstract])) OR (Leukocyte, Mononuclear[title/abstract])) OR (Peripheral Blood Mononuclear Cells[title/abstract])) OR (Bone marrow derived mononuclear cells[title/abstract])))**

**2.Cochrane**

**#1 (Monocytes OR mononuclear cells OR Mononuclear Leukocytes OR Mononuclear Leukocyte OR Leukocyte, Mononuclear OR Peripheral Blood Mononuclear Cells OR Bone marrow derived mononuclear cells):ti,ab,kw**

**#2 (Femur Head Necroses OR Head Necrosis, Femur OR Necrosis, Femur Head OR Aseptic Necrosis of Femur Head OR Necrosis, Aseptic, of Femur Head OR Necrosis, Avascular, of Femur Head OR Ischemic Necrosis Of Femoral Head OR Femoral Head, Avascular Necrosis Of OR Avascular Necrosis Of Femoral Head, Primary OR Avascular Necrosis of Femur Head):ti,ab,kw**

**#3 #2 AND #1**

**3.EMBASE**

**('femur head necroses' OR 'head necrosis，femur' OR (('head'/exp OR head) AND necrosis，femur) OR 'aseptic necrosis of femur head' OR (aseptic AND ('necrosis'/exp OR necrosis) AND of AND ('femur'/exp OR femur) AND ('head'/exp OR head)) OR 'necrosis, aseptic, of femur head' OR (('necrosis,'/exp OR necrosis,) AND aseptic, AND of AND ('femur'/exp OR femur) AND ('head'/exp OR head)) OR 'necrosis, avascular, of femur head' OR (('necrosis,'/exp OR necrosis,) AND avascular, AND of AND ('femur'/exp OR femur) AND ('head'/exp OR head)) OR 'ischemic necrosis of femoral head' OR (ischemic AND ('necrosis'/exp OR necrosis) AND of AND femoral AND ('head'/exp OR head)) OR 'femoral head, avascular necrosis of' OR (femoral AND ('head,'/exp OR head,) AND avascular AND ('necrosis'/exp OR necrosis) AND of) OR 'avascular necrosis of femoral head, primary' OR (avascular AND ('necrosis'/exp OR necrosis) AND of AND femoral AND ('head,'/exp OR head,) AND primary) OR 'avascular necrosis of femur head' OR (avascular AND ('necrosis'/exp OR necrosis) AND of AND ('femur'/exp OR femur) AND ('head'/exp OR head))) AND ((mononuclear AND cells OR peripheral) AND blood AND mononuclear AND cells OR bone) AND marrow AND derived AND mononuclear AND cells**

**4.Web of science**

**#11 #9 AND #10**

**#10 TS= (Femur Head Necroses OR Head Necrosis, Femur OR Necrosis, Femur Head OR Aseptic Necrosis of Femur Head OR Necrosis, Aseptic, of Femur Head OR Necrosis, Avascular, of Femur Head OR Ischemic Necrosis Of Femoral Head OR Femoral Head, Avascular Necrosis Of OR Avascular Necrosis Of Femoral Head, Primary OR Avascular Necrosis of Femur Head)**

**#9 TS= (mononuclear cells OR Peripheral Blood Mononuclear Cells OR Bone marrow derived mononuclear cells)**
