# Supplementary material for: Core decompression combined with bone marrow mononuclear cells in the treatment of femoral head necrosis: a systematic review and meta-analysis
Source: Int J Surg. 2024 Jul 11;110(10):6763–70. doi: 10.1097/JS9.0000000000001625 (PMC11487039; doi:10.1097/JS9.0000000000001625)
Supplement: SUPPLEMENTARY MATERIAL [file js9-110-6763-s003.docx]

| **Study** | **Reason for exclusion** |
| --- | --- |
| **[**[1](#_ENREF_1)**]** | **Inappropriate interventions** |
| **[**[2](#_ENREF_2)**]** | **Inappropriate interventions** |
| **[**[3](#_ENREF_3)**]** | **Inappropriate interventions** |
| **[**[4](#_ENREF_4)**]** | **Inappropriate interventions** |
| **[**[5](#_ENREF_5)**]** | **Inappropriate interventions** |
| **[**[6](#_ENREF_6)**]** | **Inappropriate interventions** |
| **[**[7](#_ENREF_7)**]** | **Inappropriate patient** |
| **[**[8](#_ENREF_8)**]** | **Inappropriate patient** |
| **[**[9](#_ENREF_9)**]** | **Inappropriate purpose of the study** |
| **[**[10](#_ENREF_10)**]** | **Inappropriate purpose of the study** |
| **[**[11](#_ENREF_11)**]** | **Inappropriate purpose of the study** |
| **[**[12](#_ENREF_12)**]** | **Inappropriate purpose of the study** |
| **[**[13](#_ENREF_13)**]** | **Inappropriate purpose of the study** |
| **[**[14](#_ENREF_14)**]** | **Inappropriate type of study** |
| **[**[15](#_ENREF_15)**]** | **Inappropriate type of study** |
| **[**[16](#_ENREF_16)**]** | **Inappropriate type of study** |
| **[**[17](#_ENREF_17)**]** | **Include other procedure** |
| **[**[18](#_ENREF_18)**]** | **Include other procedure** |
| **[**[19](#_ENREF_19)**]** | **Include other procedure** |
| **[**[20](#_ENREF_20)**]** | **Incomplete data** |
| **[**[21](#_ENREF_21)**]** | **Incomplete data** |
| **[**[22](#_ENREF_22)**]** | **Incomplete data** |

Reference:

1. Mao Q, Jin H, Liao F, Xiao W, Chen D, Tong P. The efficacy of targeted intraarterial delivery of concentrated autologous bone marrow containing mononuclear cells in the treatment of osteonecrosis of the femoral head: A five year follow-up study. BONE 2013;**57**(2):509-16 doi: 10.1016/j.bone.2013.08.022.

2. Cai J, Wu Z, Huang L, et al. Cotransplantation of Bone Marrow Mononuclear Cells and Umbilical Cord Mesenchymal Stem Cells in Avascular Necrosis of the Femoral Head. TRANSPLANTATION PROCEEDINGS 2014;**46**(1):151-55 doi: 10.1016/j.transproceed.2013.06.021.

3. Wu Y, Zhang C, Wu J, Han Y, Wu C. A Study of Avascular Necrosis of the Femoral Head Using Danshen Combined with Marrow Stromal Cells <i>In Vitro</i>. JOURNAL OF BIOMATERIALS AND TISSUE ENGINEERING 2019;**9**(6):845-51 doi: 10.1166/jbt.2019.2044.

4. Fu Q, Tang N-N, Zhang Q, et al. Preclinical Study of Cell Therapy for Osteonecrosis of the Femoral Head with Allogenic Peripheral Blood-Derived Mesenchymal Stem Cells. YONSEI MEDICAL JOURNAL 2016;**57**(4):1006-15 doi: 10.3349/ymj.2016.57.4.1006.

5. Rastogi S, Sankineani SR, Nag HL, et al. Intralesional autologous mesenchymal stem cells in management of osteonecrosis of femur: a preliminary study. Musculoskeletal surgery 2013;**97**(3):223-8 doi: 10.1007/s12306-013-0273-0.

6. Zhao DW, Lu FQ, Wang WM, Cui DP, Wang BJ, Zhou J. AUTOLOGOUS BONE MARROW-DERIVED AND CULTURED MESENCHYMAL STEM CELL THERAPY FOR AVASCULAR NECROSIS OF THE FEMORAL HEAD. VOX SANGUINIS 2012;**103**:263-63.

7. Hernandez A, Nunez JH, Sallent A, Gargallo-Margarit A, Gallardo-Calero I, Barro V. Core Decompression Combined with Implantation of Autologous Bone Marrow Concentrate with Tricalcium Phosphate Does Not Prevent Radiographic Progression in Early Stage Osteonecrosis of the Hip. CLINICS IN ORTHOPEDIC SURGERY 2020;**12**(2):151-57 doi: 10.4055/cios19033.

8. Daltro GC, Fortuna V, de Souza ES, et al. Efficacy of autologous stem cell-based therapy for osteonecrosis of the femoral head in sickle cell disease: a five-year follow-up study. STEM CELL RESEARCH & THERAPY 2015;**6** doi: 10.1186/s13287-015-0105-2.

9. Zhao Z. Bone Regeneration Therapy of Atraumatic Necrosis of Femoral Head. INDIAN JOURNAL OF PHARMACEUTICAL SCIENCES 2020;**82**:100-07 doi: 10.36468/pharmaceutical-sciences.sp1.67.

10. Aggarwal A, Aggarwal AK, Jha L, Arora K, Prakash M. Role of Bone Marrow derived stem cells in Avascular Necrosis of Femoral Head. MOLECULAR BIOLOGY OF THE CELL 2018;**29**(26).

11. Mao Q, Wang W, Xu T, et al. Combination Treatment of Biomechanical Support and Targeted Intra-arterial Infusion of Peripheral Blood Stem Cells Mobilized by Granulocyte-Colony Stimulating Factor for the Osteonecrosis of the Femoral Head: A Randomized Controlled Clinical Trial. JOURNAL OF BONE AND MINERAL RESEARCH 2015;**30**(4):647-56 doi: 10.1002/jbmr.2390.

12. Ma H-y, Ma N, Liu Y-f, et al. Core Decompression with Local Administration of Zoledronate and Enriched Bone Marrow Mononuclear Cells for Treatment of Non-Traumatic Osteonecrosis of Femoral Head. ORTHOPAEDIC SURGERY 2021;**13**(6):1843-52 doi: 10.1111/os.13100.

13. Zhao D, Cui D, Wang B, et al. Treatment of early stage osteonecrosis of the femoral head with autologous implantation of bone marrow-derived and cultured mesenchymal stem cells. BONE 2012;**50**(1):325-30 doi: 10.1016/j.bone.2011.11.002.

14. Xu Y, Jiang Y, Xia C, Wang Y, Zhao Z, Li T. Stem cell therapy for osteonecrosis of femoral head: Opportunities and challenges. REGENERATIVE THERAPY 2020;**15**:295-304 doi: 10.1016/j.reth.2020.11.003.

15. Li R, Lin Q-X, Liang X-Z, et al. Stem cell therapy for treating osteonecrosis of the femoral head: From clinical applications to related basic research. STEM CELL RESEARCH & THERAPY 2018;**9** doi: 10.1186/s13287-018-1018-7.

16. Cabrolier J, Molina M. [Is instillation of bone marrow stem cells at the time of core decompression useful for osteonecrosis of the femoral head?]. Medwave 2016;**16 Suppl 1**:e6406-e06 doi: 10.5867/medwave.2016.6406.

17. Kang P, Xie X, Tan Z, et al. Repairing defect and preventing collapse of femoral head in a steroid-induced osteonecrotic of femoral head animal model using strontium-doped calcium polyphosphate combined BM-MNCs. JOURNAL OF MATERIALS SCIENCE-MATERIALS IN MEDICINE 2015;**26**(2) doi: 10.1007/s10856-015-5402-x.

18. Liu Y-s, Liu S-b, Zhou S-g, et al. [Efficacies of bone-marrow-derived mononuclear cells with a hydroxylapatite composite in the treatment of osteonecrosis of the femoral head]. Zhonghua yi xue za zhi 2013;**93**(27):2126-30.

19. Yamasaki T, Yasunaga Y, Ishikawa M, Hamaki T, Ochi M. Bone-marrow-derived mononuclear cells with a porous hydroxyapatite scaffold for the treatment of osteonecrosis of the femoral head A PRELIMINARY STUDY. JOURNAL OF BONE AND JOINT SURGERY-BRITISH VOLUME 2010;**92B**(3):337-41 doi: 10.1302/0301-620X.92B3.22483.

20. Chotivichit A, Korwutthikulrangsri E, Auewarakul C, Sarirasririd S. Core decompression and concentrated autologous bone marrow injection for treatment of osteonecrosis of the femoral head. Journal of the Medical Association of Thailand = Chotmaihet thangphaet 2012;**95 Suppl 9**:S14-20.

21. Yamasaki T, Yasunaga Y, Terayama H, et al. Transplantation of bone marrow mononuclear cells enables simultaneous treatment with osteotomy for osteonecrosis of the bilateral femoral head. MEDICAL SCIENCE MONITOR 2008;**14**(4):CS23-CS30.

22. Wang T, Wang W, Yin ZS. Treatment of osteonecrosis of the femoral head with thorough debridement, bone grafting and bone-marrow mononuclear cells implantation. European journal of orthopaedic surgery & traumatology : orthopedie traumatologie 2014;**24**(2):197-202 doi: 10.1007/s00590-012-1161-2.
