# Supplementary material for: Core decompression combined with bone marrow mononuclear cells in the treatment of femoral head necrosis: a systematic review and meta-analysis
Source: Int J Surg. 2024 Jul 11;110(10):6763–70. doi: 10.1097/JS9.0000000000001625 (PMC11487039; doi:10.1097/JS9.0000000000001625)

**Supplementary Material S4:** Quality assessment and publication bias evaluation of included study.

**Table 1:** The Randomized Trials were based on the NOS quality assessment form.

| **Study** | **Random Sequence**  **Generation** | **Allocation Concealment** | **Blinding of**  **Subject/Personnel** | **Blinding Outcomes**  **Assessment** | **Attrition** | **Selective Reporting** | **Overall** |
| --- | --- | --- | --- | --- | --- | --- | --- |
| Gangji, Valérie MD  2005 | Unclear | High | High | Low | Low | Low | High |
| Ramesh Kumar Sen  2011 | Unclear | High | Unclear | Low | High | Low | High |
| Tabatabaee RM  2015 | Unclear | High | High | High | Low | Low | High |

**Table 2:** The case series studies were based on the NOS quality assessment form.

| **Study** | **Representativeness of the sample** | **Sample size** | **Selection** | **Ascertainment of exposure** | **Confounding factors are controlled** | **Assessment of the outcome** | **Statistical test** |
| --- | --- | --- | --- | --- | --- | --- | --- |
| Dawei Liang  2023 | √ | √ | √ | √ | √ | √ | √ |
| Lihua Liu  2018 |  | √ | √ | √ | √ | √ | √ |
| Yaosheng Liu  2013 | √ | √ | √ | √ | √ | √ | √ |
| Zuoqin Yan  2006 |  | √ | √ | √ | √ | √ | √ |

**Table 3:** The cohort study was based on the NOS quality assessment form.

| **Author** | **Representativeness of exposed cohort** | **Selection** | **Ascertainment of exposure** | **Demonstration that outcome of interest was not present at the start of study** | **Study control for IBLA** | **Study control for Traditional surgery** | **Assessment of outcome** | **Was follow-up long enough for outcomes to occur** | **Adequacy of follow up of cohorts** |
| --- | --- | --- | --- | --- | --- | --- | --- | --- | --- |
| Bai-Liang Wang  2009 | √ | √ | √ | √ | √ | √ | √ | √ | √ |

Reference：

**Table 4:** GRADE evaluation of outcome indicators


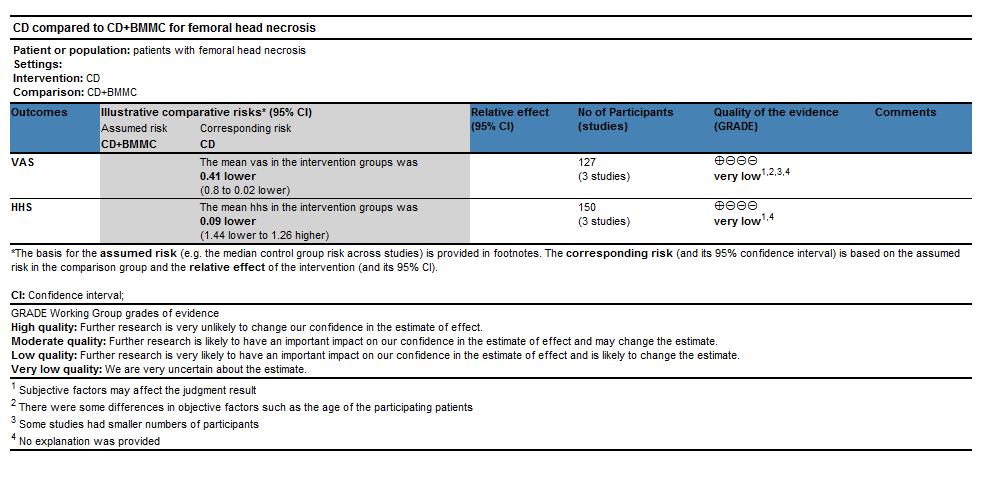

Supplement: SUPPLEMENTARY MATERIAL [file js9-110-6763-s004.docx]
